# Reply to comment on “which fraction of stone wool fibre surface remains uncoated by binder? A detailed analysis by time-of-flight secondary ion mass spectrometry and X-ray photoelectron spectroscopy” by Hirth *et al.*, 2021, *RSC Adv.*, 11, 39545, DOI: 10.1039/d1ra06251d”

**DOI:** 10.1039/d3ra02232c

**Published:** 2023-07-12

**Authors:** Sabine Hirth, Wendel Wohlleben, Hubert Waindok

**Affiliations:** a Dept. Materials Physics and Analytics, BASF SE 67056 Ludwigshafen Germany

## Abstract

This is a reply to the Comment of Okhrimenko *et al.* in the same issue of *RSC Advances*. We discuss the arguments brought forward by said authors, oppose their objections and show the unchanged validity of our results.
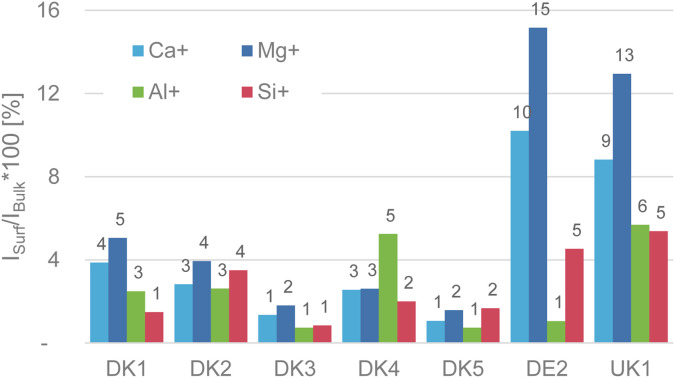

We thank the authors of the comment for their elaborate analysis of our publication and take the opportunity to reply. First, we are glad that Okhrimenko *et al.* state themselves that the findings of our publication are in line with other previously published results on stone wool samples, including results by the authors of the comment. Therefore, it is unclear to us why the authors are questioning the findings of the publication. We will reply to the technical arguments and will then reply to the conclusions with utmost brevity.

For the imaging of the distribution of organic matter (sizing) on inorganic fibres, and for our approaches to the quantification of the coverage, knowledge on the exact composition of the sizing would not have changed any of the conclusions drawn in our publication about the sizing distribution. Studies with systematically varied sizing composition could generate guidance on the composition of sizing that has the least influence on the fibre dissolution. Instead, we used field samples of MMVF that are common on the market.

Okhrimenko *et al.* suspect that the presence of organic matter on the fibre surface is an artifact of sample handling and storage. As no specific precautions against artifacts from handling and storage are noted in the publications on fibre sizing by the authors of the comment,^1–5^ we deem this argument not valid. Additionally, even thermally desized fibres which would be expected to adsorb carbon much more readily from the environment than the sized fibres show carbon concentrations well below 20% (an equivalent of 0.6 nm as calculated by the approach of Smith *et al.*^6^) when stored for prolonged times in the lab. Hence, we can safely conclude that the carbon on the fibres stems indeed from the sizing. One notes that Okhrimenko *et al.* presented results in their analysis (Fig. 1 of the comment) that are consistent with ours.^7^

**Fig. 1:**
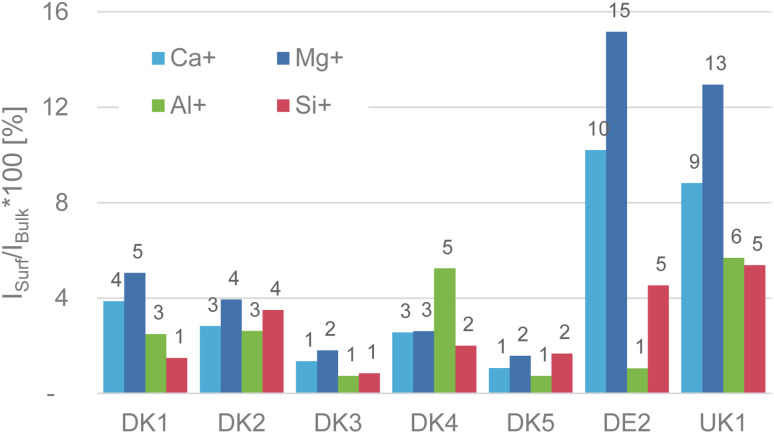
(Reproduction of Fig. 4 of the publication) Intensity ratio in percent of the elements calcium, magnesium, aluminium, and silicon on the surface referenced to their respective bulk intensity that was obtained after GCIB-sputtering to remove the sizing. For even more clarity we added the value above the bars in the chart.

Regarding the evaluation of the XPS data, we do not follow the argumentation on the Tougaard-analysis of the fibres that is shown in Fig. 2 of the comment. The choice of the range to look at the fit quality is somewhat arbitrary. The visual inspection shows that both fits exhibit the same quality around 1250 eV, 1300 eV, 1360 eV, and 1400 eV, and that fit (a) gives better fit quality in the entire range of at 1280 eV to 1330 eV and that fit (b) only gives a slightly better fit quality in the range of around 1280 eV to 1290 eV. Therefore, we must conclude that the Tougaard analysis might not be able to discriminate safely between the two scenarios on the fibres and that, even though the RMS is seemingly lower for the island model, this is a consequence of the selection of the region to calculate the RMS. The argumentation of a too low S/N of the survey spectra contradicts the statement of the QUASES-Software Manual whereby Sven Tougaard himself stated: “The application of QUASES for analysis of practical spectra is illustrated. It is found that a detailed quantification of the surface can be done even from noisy survey spectra.”^8^ Hence, the findings of the analysis should by valid even with noisy survey spectra.

**Fig. 2:**
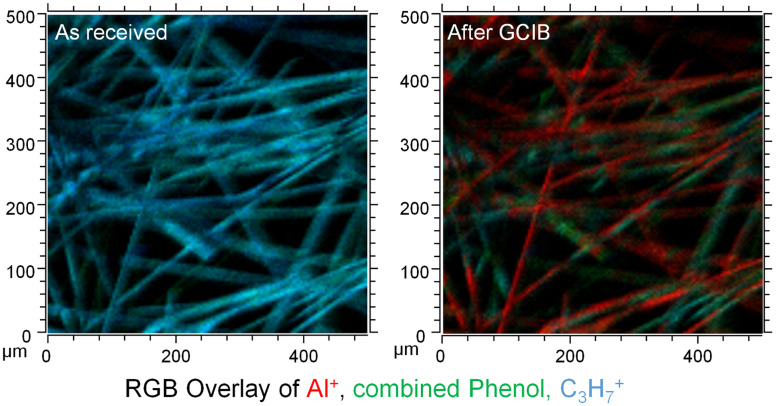
Excerpt of the image overlay for sample DE2 (originally published as part of Fig. 6 of the publication) with identical scaling of Al^+^, combined phenol and C_3_H_7_^+^ used for as received and after GCIB.

It is not convincing why the presence of 30–50 nm big droplets in any way hamper a Tougaard analysis of the Silicon and Aluminium signal. A droplet of 30–50 nm is simply “hiding” portions of the Al and Si-signal from the resulting spectra due to the limited depth of information of XPS and hence, a fit of the remaining signal would mean that the modelling of the surface as 20% “uncovered” as in Fig. 2b of the Comment would grossly overestimate the amount of uncovered material. Therefore, any presence of droplets would even increase the surface coverage quite opposite to what the authors of the comment seemingly want to imply. However, of course we also evaluated an island model for our samples but did not find a significant deviation from the buried layer model and much more stable fit results for a buried layer. Even when we resort to the other extreme and would assume that all carbon and nitrogen contribution is confined in islands thicker than the depth of information of XPS, we end up in a degree of coverage from the XPS-spectra in the range of 79 to 89% by simply adding up the amounts of carbon and nitrogen (not even taking the organic oxygen portion into account), so the finding of 20% bare surface is in principle possible but not likely in conjunction with the shape of the background behind the Si-peak.

The comment also tries to cast doubt on our results by the demand to show survey spectra, thereby implying that we had not shown relevant data. Instead, we presented with Fig. 1 of the original publication the results of the quantification of the survey spectra and we clearly state, what additional elements were present: These amounted to a maximum of 4.7 atom percent for UK1 as sum of the elements of calcium, sodium, sulphur, and magnesium in the photoelectron spectra.^7^

The weakest point of the comment is that Okhrimenko *et al.* give the impression that we claimed that no aluminium signal was observable on the as-received samples. This is not correct. We have a made a truly clear statement about the initial (as-received) intensity of the substrate signals coming from the various fibres in Fig. 4 of the original paper (reproduced here). As we already discussed in the original publication, the intensity of the fibre-related signals is in the range of 1–16% of the sputtered samples, with aluminium and silicon as the most relevant elements being in the range of only 1–5% intensity of that of the GCIB sputtered samples.^7^ This alone should be conclusion enough that the surface is covered in the attainable depth range of ToF-SIMS and it also fits well with the high amounts of nitrogen and carbon that we observed in the XPS-experiments.

Hence, our results in no way contradict the findings of the comment, quite on the contrary, we even quantified the amount of signal of substrate related signals that were visible on the as-received fibres. This was primarily done, because the selection of the image contrast of any image might distort the presentation of the results. We meticulously made sure that the dynamic range shown in the ion-maps for the signals of the GCIB-Sputtered samples and the as-received material were identical in Fig. 5 and 6 of the original publication.^7^ If such precautions are observed, the overlay of the images (showing here as an example for sample DE2, reproduced from Fig. 6 of the original publication) there can be no doubt that the fibres are covered by an organic overlayer before sputtering and that this overlayer is removed upon sputtering. Sputtering lays bare the formerly covered patches. This happens across the entirety of the fibres. Even though the layer might contain some “holes” that are below the limit of lateral resolution of the SIMS experiment, the comparison of Al-intensities before and after GCIB-sputtering proves that the surface is indeed covered heavily and that this covering material is removed by GCIB-sputtering.

Based on the above, we also need to reply shortly to Fig. 1 of the comment by Okhrimenko *et al.* Their statement that the signal from the fibre substrate dominates, cannot be safely drawn from the SIMS image of a coated sample alone as it is presented in this said Fig. 1 without knowing the ionization probabilities of the various species and the applied dynamic range of the maps. A mere ion intensity map without a proper reference (in our case the reference was the GCIB-sputtered fibre) is not sufficient to evaluate the coverage. This is shown in an example in Fig. 3, where we plotted the exact same chemical species as Okhrimenko *et al.* two times. The only difference between the two rows of images is the dynamic range that is used in the Al^+^ ion map. This gives the false impression that big parts of the substrate are uncovered.

**Fig. 3:**
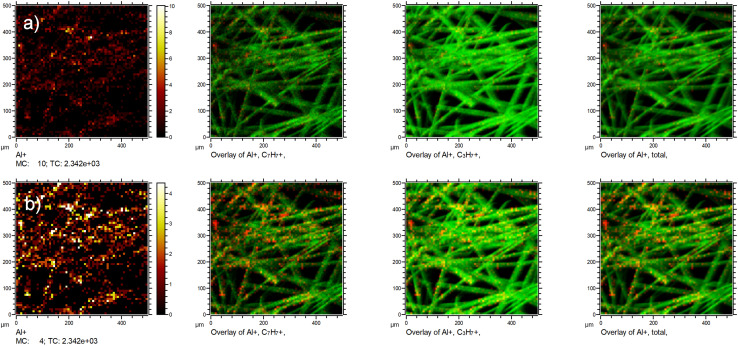
Presentation of the data for DE2 with different dynamic ranges: (a) upper row: Al^+^ ion mapping with a dynamic range in the map set to 0–10 counts per pixel (autoscale range). This map was then overlayed with autoscaled ion maps of C_7_H_7_^+^ and C_3_H_7_^+^ as well as total ion intensity. Lower row (b) Al^+^ contrast enhanced by using a dynamic range from 0–4 counts per pixel. This map was then overlayed with the identical ion maps of C_7_H_7_^+^, C_3_H_7_^+^ and total ion intensity as in row (a). This increase in image contrast leads to the false impression that big patches of uncovered fibre are present.

Hence, without referencing of intensities to the unsized surfaces as we have done with the GCIB-sputtering, it is impossible to evaluate whether the visibility of the element in the mapping is a mere artifact of the contrast used in the images as we have shown in row (b) of Fig. 3. Usually, ToF-SIMS imaging software uses an autoscaling feature to ensure maximum contrast for better visibility of features to the analyst. A careful and comparable scaling is necessary to prevent artifacts in the data presentation. As Fig. 1 of the comment neither shows a scale bar with the used dynamic range nor an indication of total ion intensity for the signals in question, and certainly no referencing to the unsized fibre intensities, it is not a sufficient presentation of data but be misleading.

However, despite these shortcomings on the evaluation, the maps in Fig. 1 of the comment show qualitatively the exact same result that we also published, and -in our view-supports our conclusion: the binder and the oil are distributed across the whole fibre, covering not only the contact points but all areas in-between. This finding must be drawn from the Bi^+^ and Bi_3_^++^ mappings alike, even though the signal of the Al^+^ from the substrate might be enhanced by using Bi^+^ as primary ion source due to changes in the ionization probabilities.

Finally, the comment repeatedly imputes that we had misunderstood or misrepresented the applicable regulation of MMV in Europe. This is not correct. The comment correctly states that current regulation in Europe relies on *in vivo* testing of fibres produced without binder. We stated the same, and added our view that the assessment by inhalation studies should cover the product as placed on the market, *i.e.* with binder. We acknowledge that technical challenges as cited by the comment must be surmounted, and exactly for this reason it is important to use complementary methods -such as biosolubility- to assess the systematic trends between MMVF without and with binder, or to devise innovative binders in a safe-and-sustainable-by-design context.^9^ There is no fundamental scientific reason to assume “that binder presence cannot affect the dissolution”, as opined by the comment. We agree on the fact that “inorganic chemical composition is among the prime factors”, but why would this preclude the modulation by additional prime factors? This question should be answered by *in vivo* studies, as required by regulation.

## Conflicts of interest

All authors are employees of BASF SE, a competitor in the market of insulation materials.

## Supplementary Material
